# A Structured Ultrasound‐Guided Cannulation Course to Prepare Medical Students for Foundation Training

**DOI:** 10.1111/tct.70320

**Published:** 2025-12-12

**Authors:** Edward Finch, Jun Jie Lim, Samuel Birks, Ansaam El‐Sherif, Stephen Cross, Jamie Sterland, Deborah Clark

**Affiliations:** ^1^ Division of Clinical Medicine, School of Medicine and Population Health The University of Sheffield Sheffield UK; ^2^ Sheffield Teaching Hospitals NHS Foundation Trust Sheffield UK

## Abstract

**Background:**

Ultrasound‐guided peripheral intravenous cannulation (US‐PIVC) is a critical skill for resident doctors, yet standardised ultrasound training remains inconsistent in undergraduate medical curricula. This gap may compromise patient care and safety.

**Approach:**

A structured, competency‐based US‐PIVC simulation training was integrated into the final‐year medical curriculum. Using the Reach, Effectiveness, Adoption, Implementation and Maintenance (RE‐AIM) framework, we conducted a convergent parallel mixed‐method study. Quantitative data were collected through a validated rating scale in an end‐of‐session assessment, whereas qualitative insights were gathered via focus group discussions.

**Evaluation:**

Ninety‐eight students participated in the simulation training, with students (*n* = 25) and staff (*n* = 4) contributing to focus group discussions. The objective competency assessment demonstrated a 98% pass rate, with 84% achieving full procedural proficiency. Thematic analysis revealed that structured US‐PIVC training significantly enhanced students' confidence and preparedness for their foundation doctor role. Participants reported a perceived reduction in dependence on senior staff and improvements in both patient safety and procedural efficacy. To ensure skill retention, key recommendations included providing ongoing practice opportunities, implementing logbook signoffs, appointing designated US skills leads, fostering collaborative partnerships and maintaining US equipment.

**Implications:**

Our study highlights the need for structured, standardised US‐PIVC training to reduce variability in clinical education. The programme improved confidence, proficiency and clinical efficiency while decreasing reliance on senior staff. Embedding mandatory training, logbook signoffs and simulation realism will enhance patient safety, procedural competency and preparedness for foundation roles.

## 1 Background

Point‐of‐care ultrasound (POCUS) has undergone major technological advances and has become the first‐line imaging modality for the clinical diagnosis of a wide range of conditions [1]. Ultrasound‐guided peripheral intravenous cannulation (US‐PIVC), in particular, is a commonly performed interventional procedure for patients with difficult intravenous access and, therefore, is an essential competency for various healthcare professionals internationally [2]. US‐PIVC training courses for doctors have demonstrated an increase in confidence and clinical utility [3] while reducing the need for more invasive venous access devices among nurses [4]. Traditionally, US‐PIVC has been highly opportunistic in the clinical setting, ranging from ‘see one, do one, teach one’ to formal, face‐to‐face courses [3]. Furthermore, the current implementation of standardised US‐PIVC training across undergraduate and postgraduate medical curricula nationally is widely variable [5]. This has resulted in low confidence and competence among healthcare professionals, compromising patient safety and timely care [6].

A systematic review suggests that simple US‐guided interventions for novices could bring about positive outcomes for patients and healthcare systems [2]. Similarly, the provision of US skills training for medical students has been proven to be well received [7]. Despite the increasing integration of US in medical school curricula in the United States and Europe, there is currently limited guidance on the implementation of a novel US‐PIVC programme nationally in the United Kingdom [8]. Furthermore, US‐PIVC educational interventions commonly face constraints such as staffing requirements and the challenge of unexpectedly detecting underlying medical conditions in volunteers, alongside broader cost–benefit concerns regarding US integration in medical education [9]. This may result in medical schools in the United Kingdom not adequately training future doctors to meet international standards, despite the importance of US‐PIVC in foundation programmes and higher specialty core training programmes [10].

In this paper, we described the implementation of a structured, competency‐based US‐PIVC training programme for medical students and evaluated its feasibility, acceptability and short‐term impact. We also assessed changes in student confidence and objectively measured competence and explored stakeholder perceptions using a mixed‐methods approach guided by an established framework.

### 1.1 Implementation Science Framework

We utilised an established implementation framework—*Reach, Effectiveness, Adoption, Implementation and Maintenance* (RE‐AIM) [11]—to enhance the replicability of the programme:

- *Reach* describes the target population and their participation.
- *Effectiveness* evaluates programme outcomes.
- *Adoption* assesses institutional uptake.
- *Implementation* examines fidelity, consistency and adaptations in programme delivery.
- *Maintenance* explores long‐term sustainability.

*We utilised an established implementation framework—Reach, Effectiveness, Adoption, Implementation and Maintenance* (RE‐AIM).

## 2 Approach

### 2.1 Course Design

**FIGURE 1:**
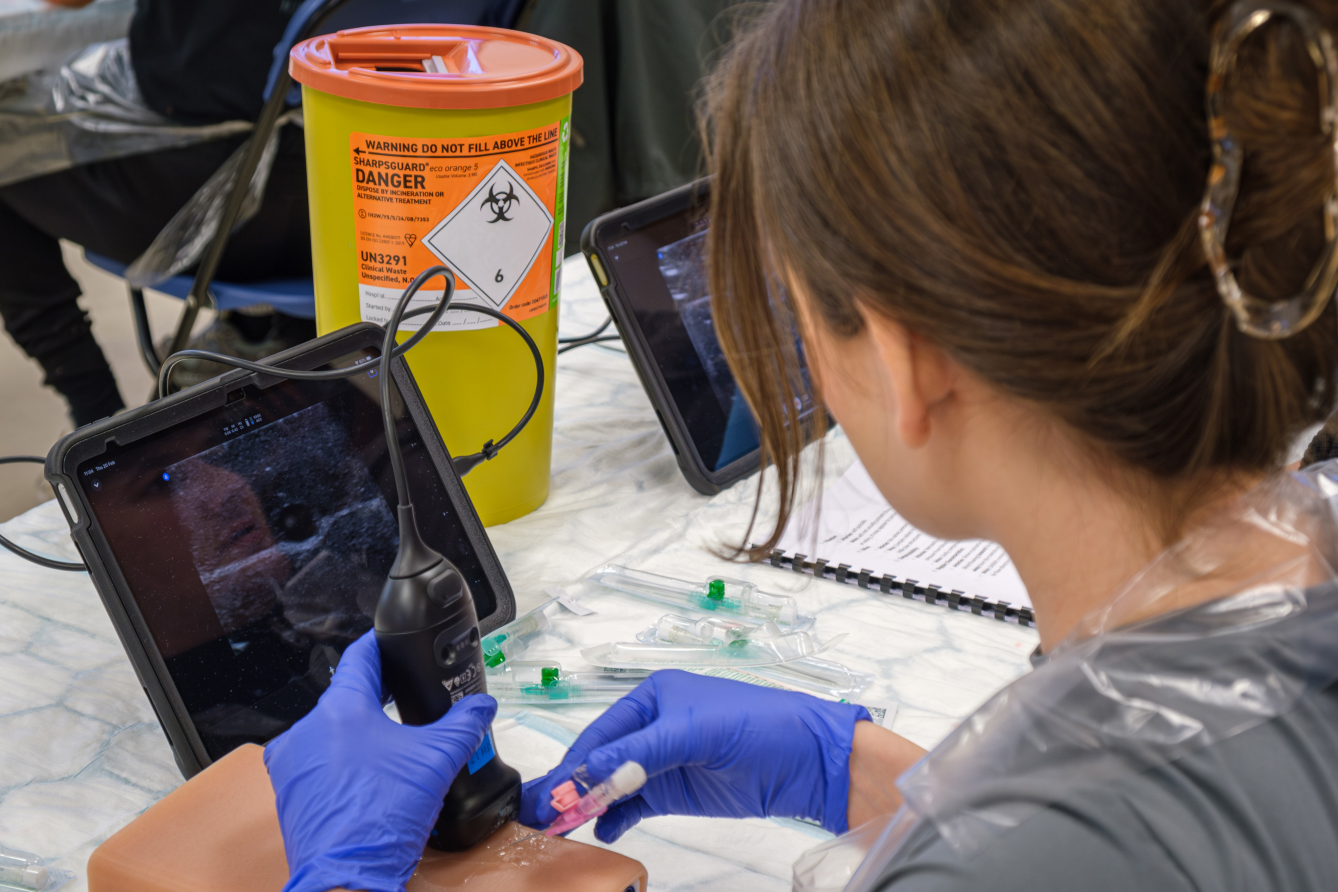
Equipment for an ultrasound‐guided peripheral intravenous cannulation course, including Butterfly iQ3 handheld ultrasound probes, iPad 10s, phantom arms, 18/20G cannulas, ultrasound gel, cleaning wipes, sharps bins and a course manual. *Note*: Figure was professionally taken by the University Photography team with written consent from participants for publication.

The US‐PIVC training comprised six sessions over three full days at the clinical skills teaching unit. Each session had a maximum capacity of 21 participants. Each student had individual access to the equipment, which included a Butterfly iQ3 handheld US probe, iPad 10, US phantom, cannulas, US gel and cleaning wipes (Figure 1). EF designed the course materials and led the programme, guided by RE‐AIM principles and literature best practices [12, 13]. The US‐PIVC training consists of:

- Asynchronous, online theory module (60 min), which included:
  ○ Teaching handbook
  ○ Step‐by‐step guide
  ○ Practical demonstration video (16 min)
  ○ Formative assessment (5 SBA questions)
- Synchronous, interactive simulation practice session (120 min), delivered by a teaching fellow leading the US, facilitated by academic foundation doctors, clinical skills educators and university academics, which included:
  ○ Theory course including image generation, equipment, probe movement, knobology and anatomy of the upper limb (45 min)
  ○ Closely supervised practice session (45 min)
  ○ 1–1 objective assessment (30 min)

We employed multiple Plan–Do–Study–Act (PDSA) cycles to guide continuous improvement through verbal and written feedback, facilitator observations, focus group discussions (FGDs) and objective student evaluations.

### 2.2 Data Collection and Analysis

A convergent parallel mixed‐method study design was adopted for data collection [14]. Quantitative data were collected through training needs assessments, and pre‐simulation and post‐simulation questionnaires, utilising 5‐point Likert scales. Students were also objectively assessed at the end of the session on a 1–1 basis on eight aspects of US‐PIVC using a validated Peripheral US‐Guided Vascular Access (P‐UGVA) Rating Scale [15], with a pass threshold score of 29/40. Qualitative data were also collected from students, educators and faculty staff members through multiple FGDs. Quantitative data were analysed using the Statistical Package for the Social Sciences (SPSS) version 29. Descriptive statistics were used to summarise the data. A *p* value of < 0.05 was considered statistically significant. The Mann–Whitney *U* test was used to compare pre‐ and post‐training confidence scores, whereas Spearman's rank correlation was used to assess the relationship between post‐training confidence scores and objective competency scores. EF and JL analysed the qualitative data using the framework method as described by Gale et al. [16], guided by the RE‐AIM framework. Transcripts were imported into NVivo (Version 15), and an initial open coding phase was undertaken independently before collaboratively developing a working analytical framework. This framework was then applied deductively across all transcripts and charted into a framework matrix to enable comparison across themes and participants. Regular team meetings were held to discuss coding consistency, refine the framework and collaboratively interpret the data to ensure rigour.

## 3 Evaluation

### 3.1 Participant Demographics

Eighty‐nine per cent (*n* = 87) of participants completed the pre‐training questionnaire, whereas 100% (*n* = 98) completed the post‐training questionnaire. Qualitative data were further analysed from four focus groups with 25 students and four faculty members. We structure our evaluation section according to the RE‐AIM implementation science framework.

### 3.2 Reach

All 126 places available were fully booked, with an attendance rate of 78% (*n* = 98), indicating high levels of interest. The programme successfully reached learners of diverse levels of clinical exposure (see Table 1).

**TABLE 1:** Participants' profile.

Characteristics		Number	Percentage
Age	21–25	74	85%
	26–30	10	11%
	> 30	3	3%
Gender	Male	29	33%
	Female	57	66%
	Non‐binary	1	1%
Prior exposure to US training	Yes	50	58%
	No	37	43%
Number of US‐PIVC observed on placement	0	18	21%
	1–5	63	72%
	> 5	6	7%
Number of US‐PIVC performed on placement	0	76	87%
	1–5	11	13%
	> 5	0	0%
Barriers to performing US‐PIVC^a^	Lack of structured training programme	55	63%
	Insufficient opportunity or time	50	58%
	Lack of equipment and physical space	32	37%
	Trainers with limited experience	13	15%
	Inadequate trainee preparation	12	14%

^a^ Multiple responses allowed.

Within the FGDs, students highlighted two primary barriers to participation: limited availability of training slots and scheduling conflicts. Despite these challenges, students expressed strong interest in attending the programme and suggested that integrating training into existing clinical rotations, such as anaesthetics, would improve accessibility (see Table 2).

**TABLE 2:** Subthemes and illustrative quotations of focus group discussions.

Subtheme	Quotes
Reach	
Limited availability	‘There are limited spaces so not everyone on the course could do it if they wanted to, but they added more slots in because we have got a huge cohort’ (Student 19)
Timing conflicts	‘It's quite close to finals, so you might be less willing to come to additional teaching sessions that's not directly relevant for exams’ (Student 12)
Integration into clinical rotations	‘If it was scheduled for a mandatory part, maybe making it part of our anaesthetic rotation, that would probably be better because you know you are coming and you are allowed to come’ (Student 5)
Effectiveness	
Improved confidence	‘I tried doing some Ultrasound (US)‐guided cannulations and I really struggled. I now find cannulating using US a lot easier, and I feel I understand the principles of using US that I can use for other procedures. It is useful to know before graduating rather than being expected to know later on the job’ (Student 3)
Improved preparedness for the foundation doctor role	‘This is such a valuable skill for foundation training, and I feel relieved to have had some training before starting work and night shifts as the lone doctor dealing with difficult cannulation requests. Often, I've seen F1's be left with tricky cannulations and struggle to do so, this will be a huge benefit to know and be confident to try’ (Student 22)
Reduced dependence on seniors	‘It will help me deal with difficult scenarios on the ward and being able to try cannulating with US independently before escalating it to someone more senior’ (Student 9)
Benefiting patient care	‘Have encountered many situations where staff were waiting for anaesthesia, delaying patient care, using US to do this allows fewer attempts for the patient, reduce patient discomfort so this skill will be valuable’ (Student 18)
Desire for earlier training	‘Wish we had this training in medical school sooner. This would have been very useful earlier and would have helped us practice US cannulas on the wards. With POCUS (Point‐of‐care US) coming into a lot of other med school curricula, I feel it would be very useful for medical students to have teaching on US cannulation’ (Student 5)
Adoption	
Standardisation	‘US teaching has been so variable all over the different trusts. Personally, I cannot actually remember any formal US teaching … sometimes it varies depending on hospitals as well, it's not consistent everywhere, so this training makes sure that everyone could have it’ (Student 16)
Curricular embedding	‘I think this should be integrated as a mandatory training as part of our A&E (Accident and Emergency) or anaesthetic block earlier in the course because then we can have more opportunities to implement it in practice once we have learned it’ (Student 1)
Ensuring adequate resources	‘I think it's very exciting. It's been a long time coming, and I'm very glad we have actually got everything together now, the knowledge, the clinical experience, the equipment, and dedicated people who have pushed US‐PIVC forward’ (Faculty 2)
Programme expansion	‘We can either expand who we teach cannulation to, or we can expand the concept of US to use it in different areas such as anatomy or using US equipment to teach examination skills. I suppose with the physician assistants definitely, and for nursing as well’ (Faculty 1)
Implementation	
Good balance of theory and practice	‘I think previous teaching has been brief, it's more like you just taught what to do, and you just do it on a whim, you do not actually know what you are looking for or why you are doing certain things. For this teaching, I understand the theory and how to do things’ (Student 21)
Individual access to equipment	‘I've had a session before, and everyone was crowded around watching. There are lots of US probes this time, so each person got their own US machine and arm, so all got to have a go at the same time, no waiting around’ (Student 4)
Supportive tutors	‘I think this is the optimal setting because we do not really get supervised teaching in the wards. I think having someone, at all times, telling you like tips, and having someone watch you to make sure your technique is good, which is like it was today, is good’ (Student 23)
Organisational consistency	‘I think it worked well because we maintained consistency, we have consistent trainers, and the teaching was delivered in the same way everytime. I think a consistent approach is very important in training a cohort of students, so that they all receive the same information in a similar fashion’ (Faculty 2)
Enhance realism	‘I think it'd be great in future sessions to bring a bit of reality into it with helping students develop confidence in explaining the procedure to simulated patients. Practicing on phantom arms are useful, but there's also a lot of communication involved in the real clinical environment’ (Faculty 1)
Maintenance	
Continual practice opportunities	‘It would be useful to have the opportunity to self‐practice in drop‐in sessions, so you can actually book into a session to practice yourself’ (Student 14)
Logbook signoffs	‘We've done all this excellent teaching and when these students go out there into clinical practice, we could sign it off as what we do with the skills here, just keeping them up‐skilled. That's the role of the logbook’ (Faculty 3)
Designated US skills lead	‘The mannequins we are using need resetting, and that would have to be built into a job role and session timing’ (Faculty 1)
Collaborative partnerships	‘The plan moving forward is to collaborate with partnering hospitals to increase faculty and manpower to deliver teaching on a larger scale’ (Faculty 4)
Equipment sustainability	‘Technology goes out of date very quickly … it's about how we keep up to date with all of that’ (Faculty 3)

### 3.3 Effectiveness

Overall, 98% (*n* = 96) of participants passed the end‐of‐session objective US‐PIVC competency assessment, with 84% (*n* = 82) completing all aspects of the US‐PIVC procedure in full. Students demonstrated a statistically significant improvement in confidence across all measured learning outcomes following the training (*p* < 0.001). Notably, the effect sizes for these improvements were large across all outcomes (*r* = 0.63–0.87), indicating substantial practical significance in addition to statistical significance (see Table 3). However, there was no significant correlation between students' post‐training confidence ratings and their objectively assessed competency scores (*p* = 0.969), indicating that although students felt more confident, this did not predict performance in the assessment. Framework analysis revealed five key themes within the effectiveness domain: (1) ‘improved confidence’, (2) ‘improved preparedness for the foundation doctor role’, (3) ‘reduced dependence on seniors’, (4) ‘benefiting patient care’ and (5) ‘desire for earlier training’ (see Table 2). Participants reported increased confidence in using US for cannulation and highlighted how the session improved their preparedness for the foundation doctor role. Many described reduced dependence on senior colleagues when faced with difficult cannulation scenarios going forward, contributing to a sense of autonomy and readiness. Students also felt that improved US‐PIVC skills had the potential to reduce patient discomfort and procedural delays and expressed a strong desire for earlier exposure to this training within the medical curriculum.

**TABLE 3:** Comparison of student confidence in learning outcomes pre and post the teaching session.

Learning outcomes	Pre‐teaching median^a^ (IQR)	Post‐teaching median^a^ (IQR)	*p*	Effect size (*r*)
Understanding of the theory of US image generation	2 (1–3)	4 (3–4)	< 0.001	0.69
Knowledge of the indications and contraindication	2 (1–3)	4 (4–4)	< 0.001	0.87
Understanding of infection prevention measures	3 (2–3)	5 (4–5)	< 0.001	0.70
Ability to optimise US image	1 (1–2)	4 (3–5)	< 0.001	0.80
Knowledge of relevant anatomy	3 (1–3)	4 (3–4)	< 0.001	0.67
Ability to differentiate between a vein from an artery	3 (2–4)	5 (4–5)	< 0.001	0.63
Confidence in performing US‐guided cannulation	1 (1–2)	4 (3–5)	< 0.001	0.81

Abbreviation: IQR = interquartile range.

^a^ The responses to each statement were scored using a Likert scale ranging from 1 to 5 (1 = *not confident at all*, 5 = *very confident*).

### 3.4 Adoption

Focus group data revealed significant variability in students' prior exposure to US‐PIVC training across different hospitals and trusts. This inconsistency highlighted the need for standardised, equitable access to training opportunities. Under the Adoption domain, students expressed strong support for formally integrating US‐PIVC teaching into the undergraduate curriculum. Many favoured embedding it within rotations such as anaesthetics or emergency medicine, where clinical application would be immediate and relevant. Faculty members acknowledged the programme's feasibility, citing sufficient staff and equipment availability to support wider adoption. The faculty have since expanded the teaching into the physician assistants course, demonstrating the programme's wider adoption to allied health professions education curricula (see Table 2).

### 3.5 Implementation

The programme was delivered with minimal problems and inconsistencies, as reported by 81% (*n* = 79) of participants. This was further supported by consistent objective assessment outcomes across all six sessions, with a median competency score of 40 out of 40. Framework analysis of qualitative data identified five key enablers of successful delivery: a balanced integration of theory and practical skills, individual access to US equipment, the presence of supportive tutors, organisational consistency and a desire for increased simulation realism. Students appreciated the structured, hands‐on format and valued having their own equipment to practice rather than observing passively (see Table 2). Despite these strengths, four overarching categories of inconsistencies were also identified: equipment and materials, logistics, technical aspects and instructor variability. In response, iterative changes were implemented throughout the programme via multiple PDSA cycles, such as standardised instructor briefings, improved session structuring, enhanced ergonomics teaching and stricter equipment checks (see Table 4).

**TABLE 4:** Inconsistencies identified, changes implemented and future recommendations of US‐PIVC training course.

Category	Inconsistencies identified	Changes implemented	Future recommendations
Equipment and materials	Insufficient cannula supply	Restricted usage per student	Consider increasing supply and adjusting limitations if needed
	Ultrasound probes becoming warm to touch	Rotated probes to prevent excessive warming	Explore cooling strategies and ensure adequate number of probes to allow rotation
	Spillage of simulated blood products	Mandated PPE use (gloves, aprons) and enforced bungs	Investigate alternative training fluids
	No flashback during cannulation (phantom arm pressure variations)	Ensured phantom arms were properly topped up and reassured students	Research alternative phantom arm designs for improved realism
	Equipment malfunctions (probe/iPad battery issues)	Pre‐checked equipment before sessions	Invest in additional chargers or backup devices
	Realism of US phantom	Provided real‐life clinical scenarios and examples	Incorporate more real‐world case discussions
Logistics	Insufficient practice time	Extended hands‐on practice sessions	Adjust session structure to allow more practice time
	Sessions running over time	Moved assessments 45 min before session end	Continue evaluating session timing for efficiency
Technical aspects	Poor positioning during cannulation	Emphasised proper ergonomics and phantom arm positioning	Reinforce techniques through pre‐session briefings
	Difficulty with one‐handed cannulation	Taught specific one‐handed technique	Introduce structured one‐handed cannulation drills
	Needling difficulties (over‐/under‐advancement)	Implemented targeted teaching with animation slides	Gather student feedback to refine teaching methods
	Artefacts and poor imaging clarity	Taught techniques to minimise artefacts and ensured proper use of ultrasound gel	Explore improved phantom materials
Instructor variability	Variable helpfulness of instructors	Standardised instructor briefing	Provide additional training and feedback sessions
	Inconsistent marking between examiners	Provided examiner briefing	Develop a standardised marking rubric
	Challenges with paper‐based assessments (errors, inefficiencies)	Plan to transition to iPad‐based marking	Implement a digital assessment system as soon as feasible

*Iterative changes were implemented throughout the programme via multiple PDSA cycles*.

### 3.6 Maintenance

Post‐training survey revealed that 81% (*n* = 79) of participants agree that they will continue to use and refine the US‐guided cannulation skills learned in this training for their future practice. Within focus groups, learners suggested self‐directed drop‐in sessions to reinforce skills beyond initial training. Educators highlighted the need to incorporate logbook signoffs to ensure real‐life implementation of learning and competency maintenance. The faculty proposed appointing designated roles to oversee US training. They also emphasised scaling the programme through collaborations with partnering hospitals. Both learners and educators stressed the importance of sustaining equipment technology to maintain training quality (see Table 2).

## 4 Implications

The US‐PIVC programme significantly improved students' confidence, clinical preparedness and objective competence. Its consistent delivery across six cohorts demonstrates strong feasibility and potential for scalability across the healthcare professions education continuum. These findings provide timely evidence to support the structured integration of US‐PIVC into undergraduate curricula, particularly given the increasing demand for US proficiency in foundation and specialty training.

*The US‐PIVC programme significantly improved students' confidence, clinical preparedness and objective competence*.

This study also challenged previous claims [9] that undergraduate ultrasound training lacks empirical justification. By providing both quantitative and qualitative evidence of impact, this study supports the integration of US‐PIVC training into undergraduate clinical curricula. The variability and inconsistency in existing US‐PIVC training highlight the need for structured, standardised programmes to ensure all medical students acquire this essential skill before entering clinical practice [10]. Whereas most previous research has focused on postgraduate US‐PIVC training [3], our study provides compelling evidence for its incorporation into undergraduate education, where delivery may be more consistent and logistically feasible. This is particularly relevant given ongoing challenges in postgraduate training, including limited faculty, resource constraints and variability in instructional quality [5].

*The variability and inconsistency in existing US‐PIVC training highlight the need for structured, standardised programmes*.

### 4.1 Future Suggestions

Given the rapid expansion of US training in healthcare curricula, we encourage further research into the long‐term impact of structured US‐PIVC programmes on clinical competency and patient outcomes. Incorporating follow‐up assessments into clinical placements may provide further insight into skill retention and transfer to practice. There is also scope to evaluate the role of near‐peer teaching, particularly using senior medical students to support skill development in earlier years. With proper implementation and broader integration of structured US training within undergraduate clinical curricula, this could significantly improve the preparedness of future foundation doctors and benefit patient care.

## Author Contributions

**Edward Finch:** investigation, conceptualization, writing – original draft, methodology, validation, visualization, writing – review and editing, formal analysis, project administration, data curation, supervision, resources. **Jun Jie Lim:** investigation, writing – original draft, methodology, validation, visualization, writing – review and editing, formal analysis, data curation. **Samuel Birks:** writing – review and editing, methodology, supervision, resources, validation, investigation. **Ansaam El‐Sherif:** data curation, writing – review and editing, formal analysis. **Stephen Cross:** writing – review and editing, resources, project administration. **Jamie Sterland:** writing – review and editing, resources, project administration. **Deborah Clark:** supervision, writing – review and editing, resources, project administration.

## Funding

The authors received no specific funding for this work.

## Ethics Statement

The School of Medicine and Population Health, the University of Sheffield, granted ethical approval for this research (Reference Number 064511).

## Conflicts of Interest

The authors declare no conflicts of interest.

## Supporting information


**Data S1:** Pre‐training questionnaire.
**Data S2:** Post‐training questionnaire.
**Data S3:** Peripheral Ultrasound‐Guided Vascular Access (P‐UGVA) Rating Scale.

## Data Availability

The data that support the findings of this study are available from the corresponding author upon reasonable request.

## References

[1] N. Smallwood and M. Dachsel , “Point‐of‐Care Ultrasound (POCUS): Unnecessary Gadgetry or Evidence‐Based Medicine?,” Clinical Medicine 18, no. 3 (2018): 219–224. 29858431 10.7861/clinmedicine.18-3-219 PMC6334078

[2] M. J. Hoskins , B. C. Nolan , K. L. Evans , and B. Phillips , “Educating Health Professionals in Ultrasound Guided Peripheral Intravenous Cannulation: A Systematic Review of Teaching Methods, Competence Assessment, and Patient Outcomes,” Medicine 102, no. 16 (2023): e33624. 37083799 10.1097/MD.0000000000033624 PMC10118335

[3] M. Pham , R. Aldous , and S. Brincat , “Implementation of Ultrasound‐Guided Cannulation Training for Foundation Doctors,” Clinical Medicine 24, no. 6 (2024): 100256. 39447875 10.1016/j.clinme.2024.100256 PMC11651033

[4] B. Galen , S. Baron , S. Young , A. Hall , L. Berger‐Spivack , and W. Southern , “Reducing Peripherally Inserted Central Catheters and Midline Catheters by Training Nurses in Ultrasound‐Guided Peripheral Intravenous Catheter Placement,” BMJ Quality and Safety 29, no. 3 (2020): 245–249. 10.1136/bmjqs-2019-009923 31582569

[5] L. A. Stolz , U. Stolz , C. Howe , I. J. Farrell , and S. Adhikari , “Ultrasound‐Guided Peripheral Venous Access: A Meta‐Analysis and Systematic Review,” Journal of Vascular Access 16, no. 4 (2015): 321–326. 25656255 10.5301/jva.5000346

[6] N. Horner , N. Bruce , R. Dornan , et al., “WP8.10 ‐ Ultrasound Cannulation Training Programme: Quality Improvement Project,” British Journal of Surgery 111, no. 8 (2024): 197–220.

[7] E. McCormick , B. Flanagan , C. D. Johnson , and E. M. Sweeney , “Ultrasound Skills Teaching in UK Medical Education: A Systematic Review,” Clinical Teacher 20, no. 5 (2023): e13635. 37655446 10.1111/tct.13635

[8] A. M. Armson , R. Moynihan , N. Stafford , and C. Jacobs , “Ultrasound‐Guided Cannulation for Medical Students,” Clinical Teacher 18, no. 3 (2021): 295–300. 33565228 10.1111/tct.13334

[9] Z. Feilchenfeld , T. Dornan , C. Whitehead , and A. Kuper , “Ultrasound in Undergraduate Medical Education: A Systematic and Critical Review,” Medical Education 51, no. 4 (2017): 366–378. 28118684 10.1111/medu.13211

[10] M. S. Campos , C. J. Donaldson , G. Rajeswaran , and I. Ahmad , “The Role of Ultrasound Teaching in the Undergraduate Medical Curriculum,” Clinical Teacher 16, no. 5 (2018): 539–540. 30406968 10.1111/tct.12969

[11] R. E. Glasgow , T. M. Vogt , and S. M. Boles , “Evaluating the Public Health Impact of Health Promotion Interventions: The RE‐AIM Framework,” American Journal of Public Health 89, no. 9 (1999): 1322–1327. 10474547 10.2105/ajph.89.9.1322 PMC1508772

[12] M. J. Griksaitis , M. P. Scott , and G. M. Finn , “Twelve Tips for Teaching With Ultrasound in the Undergraduate Curriculum,” Medical Teacher 36, no. 1 (2014): 19–24. 24156786 10.3109/0142159X.2013.847909

[13] L. McMenamin , F. E. Brown , M. Arora , et al., “Twelve Tips for Integrating Ultrasound Guided Peripheral Intravenous Access Clinical Skills Teaching Into Undergraduate Medical Education,” Medical Teacher 43, no. 9 (2020): 1010–1018. 33161823 10.1080/0142159X.2020.1841127

[14] J. W. Creswell and V. L. Clark , “Choosing a Mixed Methods Design,” in Designing and Conducting Mixed Methods Research, eds. J. W. Creswell and V. L. Clark (Sage, 2007), 53–71.

[15] S. C. Primdahl , J. Weile , L. Clemmesen , et al., “Validation of the Peripheral Ultrasound‐Guided Vascular Access Rating Scale,” Medicine 97, no. 2 (2018): e9576. 29480851 10.1097/MD.0000000000009576 PMC5943877

[16] N. K. Gale , G. Heath , E. Cameron , S. Rashid , and S. Redwood , “Using the Framework Method for the Analysis of Qualitative Data in Multi‐Disciplinary Health Research,” BMC Medical Research Methodology 13, no. 1 (2013): 117. 24047204 10.1186/1471-2288-13-117 PMC3848812

