# Supplementary material for: A Structured Ultrasound‐Guided Cannulation Course to Prepare Medical Students for Foundation Training
Source: Clin Teach. 2025 Dec 12;23(1):e70320. doi: 10.1111/tct.70320 (PMC12700721; doi:10.1111/tct.70320)
Supplement: Supplementary file 1 — Data S1: Pre‐training questionnaire. Data S2: Post‐training questionnaire. Data S3: Peripheral Ultrasound‐Guided Vascular Access (P‐UGVA) Rating Scale. [file TCT-23-e70320-s001.doc]

**Supplementary materials**

**Supplementation material 1: Pre-training questionnaire**

1. What is your age?
2. What is your gender?

- Male
- Female
- Non-binary
- Others: _______________

1. Do you have prior exposure to ultrasound training?

- Yes
- No

1. What are the barriers to you performing ultrasound-guided cannulation?

- Lack of structured training programme
- Insufficient opportunity or time
- Lack of equipment and physical space
- Trainers with limited experience
- Inadequate trainee preparation

1. What is the number of ultrasound-guided cannulas observed on clinical placement?
2. What is the number of ultrasound-guided cannulas performed on clinical placement?
3. Please indicate how you would rate yourself on the following on a scale of 1 (strongly disagree) to 5 (strongly agree) pre-training:

- Understanding of the theory of US image generation
- Knowledge of the indications and contraindication
- Understanding of infection prevention measures
- Ability to optimise ultrasound image
- Knowledge of relevant anatomy
- Ability to differentiate between an a vein from an artery
- Confidence in performing ultrasound guided cannulation
- Ability to confirm cannulation of a vein

**Supplementation material 2: Post-training questionnaire**

1. Please indicate how you would rate yourself on the following on a scale of 1 (strongly disagree) to 5 (strongly agree) post-training:

- Understanding of the theory of US image generation
- Knowledge of the indications and contraindication
- Understanding of infection prevention measures
- Ability to optimise ultrasound image
- Knowledge of relevant anatomy
- Ability to differentiate between an a vein from an artery
- Confidence in performing ultrasound guided cannulation
- Ability to confirm cannulation of a vein

1. Following this teaching can you tell us how this teaching would help you as a medical student/ foundation doctor?
2. Please indicate how you would rate yourself on the following on a scale of 1 (strongly disagree) to 5 (strongly agree)

- The ultrasound-guided cannulation training was delivered as planned with minimal inconsistencies
- I will continue to use and refining the US-guided cannulation skills learned in this training in your future practice

1. Please describe any inconsistencies or problems in the training that you noticed and how they impacted your learning:

**Supplementary material 3: Peripheral Ultrasound-Guided Vascular Access (P-UGVA) Rating Scale.**

| **Category** | **1** | **2** | **3** | **4** | **5** |
| --- | --- | --- | --- | --- | --- |
| **Preparation of utensils** | No preparation before scan. |  | Incomplete preparation. Unnecessary interruptions during the procedure or need for assistance. |  | Perfect preparation. Procedure performed in a smooth workflow. |
| **Ergonomics** | Working posture and apparatus positioning complicate the procedure unnecessarily. |  | Partial optimization of working posture. |  | Perfect working posture and positioning of the apparatus. |
| **Preparation of the ultrasound device** | Incorrect selection and/or orientation of transducer. No image optimization. |  | Inconsistent selection and/or orientation of transducer. Incomplete image optimization. |  | Correct selection and orientation of transducer. Image optimization performed systematically. |
| **Identification of blood vessels** | No regards to distinction between arteries and veins. No optimization of vessel filling. |  | Insecure distinction between arteries and veins. Incomplete optimization of vessel filling. |  | Perfect distinction between arteries and veins. Optimization of vessel filling. |
| **Anatomy** | Random approach to location. Important structures are neglected. Unsuitable puncture site. |  | Partially systematic approach to location of vessels. |  | Systematic location of target vessel. Recognition of all important anatomy. Most suitable puncture site. |
| **Hygiene** | Shows no regards to hygiene. |  | Follows guidelines partially. |  | Follows guidelines. |
| **Coordination of the needle** | Lack of control and navigation of the needle tip. Misses target vessel. |  | Insecure control and navigation of the needle tip. Places needle in target vessel. |  | Full control of the needle tip and navigates to perfection. Places needle in target vessel. |
| **Completion of the procedure** | Intravascular placement is not ensured. |  | Intravascular placement is ensured partially. |  | Intravascular placement is ensured correctly. |

Citation: Primdahl SC, Weile J, Clemmesen L, Madsen KR, Subhi Y, Petersen P, Graumann O. Validation of the peripheral ultrasound-guided vascular access rating scale. Medicine. 2018 Jan 1;97(2):e9576.
